# Cell biological analysis of mosquito midgut invasion: the defensive role of the actin-based ookinete hood

**DOI:** 10.1179/2047772413Z.000000000180

**Published:** 2013-12

**Authors:** Timm Schlegelmilch, Dina Vlachou

**Affiliations:** Department of Life Sciences, Imperial College London, UK

**Keywords:** Malaria, *Plasmodium*, *Anopheles gambiae*, mosquito midgut invasion, ookinete hood, host-parasite interactions

## Abstract

Successful completion of the *Plasmodium* lifecycle in the mosquito vector is critical for malaria transmission. It has been documented that the fate of *Plasmodium* in the mosquito ultimately depends on a fine interplay of molecular mosquito factors that act as parasite agonists and antagonists. Here we investigate whether the cellular responses of the invaded midgut epithelium can also determine the parasite fate and development. We show that the parasite hood, an actin-rich structure formed around the ookinete as it exits the epithelium, is a local epithelial defence reaction observed around 60% of invading parasites. The hood co-localizes with WASP, a promoter of actin filament nucleation, suggesting that it is an active reaction of the invaded cell against invading parasites. Importantly, depletion of WASP by RNAi leads to a significant reduction in hood formation, which is consistent with the previously documented role of this gene as a potent parasite antagonist. Indeed, in mosquitoes that are either genetically selected or manipulated by RNAi to be refractory to *Plasmodium*, most dead parasites exhibit an actin hood. In these mosquitoes, invading ookinetes are killed by lysis or melanization while exiting the midgut epithelium. Silencing WASP in these mosquitoes inhibits the formation of the hood and allows many parasites to develop to oocysts. These data in conjunction with fine microscopic observations suggest that the presence of the hood is linked to ookinete killing through lysis.

## Introduction

Malaria is one of the most devastating infectious diseases, threatening half of the global population. Almost half a billion people are infected every year, of which up to one million die, mostly children below five in sub-Saharan Africa. For disease transmission, the malaria parasite *Plasmodium* has to complete a complex developmental lifecycle in a female *Anopheles* mosquito. *Plasmodium* gametocytes are taken up by the mosquito with a bloodmeal and produce gametes of both sexes in the midgut lumen, which fertilize and produce zygotes that soon transform into invasive ookinetes. Ookinete traversal of the midgut epithelial cell wall is a very critical step of the parasite lifecycle. Each oocyst that develops on the basal side of the midgut upon successful ookinete invasion gives rise to thousands of sporozoites that eventually migrate to and invade the salivary glands, ready to spread the disease with consecutive mosquito bloodmeals. Studies on parasite population dynamics suggest that the ookinete-to-oocyst transition is indeed the weakest link in the entire transmission cycle, as parasite numbers often drop from hundreds down to single digits.^1^ Therefore, understanding the processes that take place during this step could guide approaches for preventing transmission.

To traverse the mosquito midgut epithelium, *Plasmodium* ookinetes initially follow a predominantly intracellular route, but they also engage into extensive lateral migration through consecutive midgut cells until they exit the epithelium into the sub-epithelial space.^2^–^4^ While migrating intracellularly, the parasites are in direct contact with the cytoplasm of the invaded cells without being surrounded by a parasitophorous vacuole.^5^ The damage inflicted to the invaded cell is irreversible and ultimately leads to apoptosis.^2^,^4^,^6^ Thereby, the cell alters morphologically with a substantial loss of microvilli^6^ and protrudes towards the apical side of the epithelium.^2^,^4^,^6^ While protrusion of the cell appears to be mediated by a purse-string mechanism at the basal side of the dying cell,^6^ the surrounding area undergoes a series of cellular responses to seal the invaded area, including neighbouring cells becoming elongated and extending lamellipodia towards the protruding cell.^4^ A lamellipodia-like structure extended by the invaded cell itself towards migrating parasites was also observed; it tightly covers ookinetes like a ‘hood’ while emerging from the epithelium into the sub-epithelial space.^4^

Morphological resemblance of the parasite hood with the ‘phagocytic cup’ that is formed by phagocytes around ingested bacteria together with the documented involvement of key parasite antagonists, such as TEP1 and LRIM1, in bacterial phagocytosis, led us to hypothesize that the parasite hood might represent an epithelial defence reaction.^4^,^7^–^10^ We have previously shown with electron micrographs that the hood is an actin-rich structure, and that silencing activators and inhibitors of actin nucleation leads to decreased and increased numbers of parasites, both the rodent model *P. berghei* and the virulent human parasite *P. falciparum*, respectively.^4^,^11^,^12^ Interestingly, ookinetes of the avian parasite *P. gallinaceum* are also surrounded by filamentous material resembling the hood in the midgut of a lytic strain of *A. gambiae*.^13^ In the same context, it has been previously documented that development of an organelle-free actin zone at the ookinete on the basal side of the epithelium depends on TEP1 presence, and this zone is linked to PPO activation and melanization.^14^ Presumably, this zone is the same as the ookinete hood.

Here we present an extensive cell-biological analysis of actin cytoskeleton dynamics in respect to invading ookinetes in the midgut epithelium. We show that the parasite hood is indeed rich in filamentous actin (F-actin) and around 60% of invading ookinetes are observed. It co-localizes with WASP, suggesting that it is generated by the invaded cell through signalling as a response to events that occur during ookinete invasion. We reveal that the antagonistic effect of *WASP* against parasites is linked to a significant reduction in hood formation, in support of our hypothesis that the hood is indeed a local epithelial defence reaction. In consistence with this hypothesis are our findings that, in the refractory ookinete-melanizing strain, most dead ookinetes also exhibit parasite hoods, and that silencing WASP allows development of some oocysts.

## Results

### The actin-rich hood is formed by the invaded cell

To determine whether the parasite hood is indeed an actin-based structure, infected *A. gambiae* midgut epithelia with the *Pb*GFP_CON_ parasite^15^ were dissected at 23–25 hours after the bloodmeal, a period that corresponds to midgut invasion, and stained with fluorophore-conjugated phalloidin, a phallotoxin that specifically binds F-actin. Using confocal microscopy, high-resolution 3D image stacks were recorded and the presence of actin staining associating with invading parasites was analysed.

**Figure 1:**
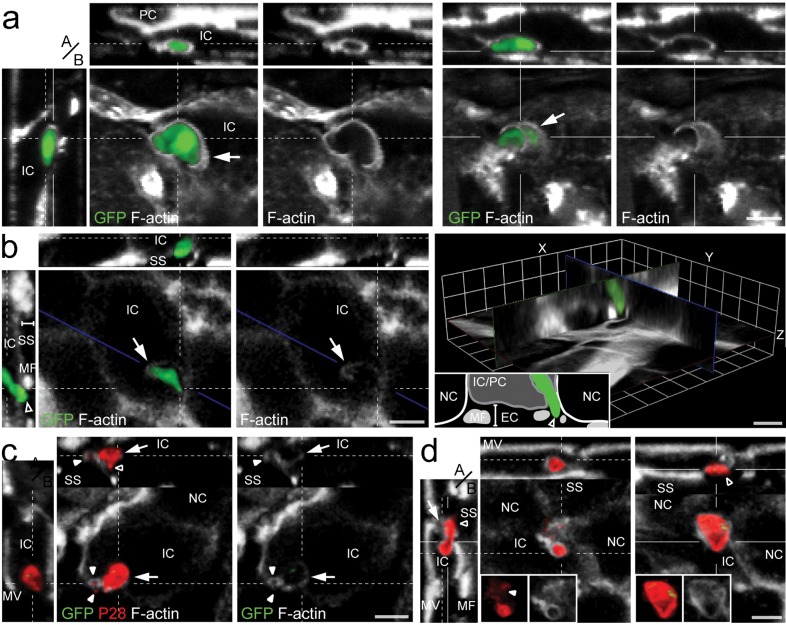
Selected 3D confocal image datasets of mosquito midguts infected with *P. berghei* GFP fluorescence parasite showing invading ookinetes surrounded by actin hood structure while exiting the epithelium. Panels show representative images and include single confocal sections in the *x*–*y* plane as well as pseudo-cross-sections in the perpendicular (apical, basal, a/b) *z* axis, which are *x*–*z* (top) and *y*–*z* (left). The *x*- and *y*-axes are always in the plane of the epithelium. The position of the pseudo-cross-sections is indicated in the main images by fine white lines crossing in the parasite image; similarly, the plane of the main image is indicated by a fine white line in each pseudo-cross-section. Parasite (green); P28 staining (red); F-actin staining with phalloidin (grey); microvilli (MV); muscle fibres (MF); invaded cells (IC); neighbour cells (NC); extracellular space (EC); cell being protruded to the midgut lumen (PC); sub-epithelial space (SS); Scale bars represent 5 *μ*m. (a) An ookinete (green) partially covered by a thick actin-rich hood (arrow) while exiting a midgut cell. A more apical view of the ookinete (dashed lines; left image set) which is fully surrounded by actin hood as well as a more basal view of the same ookinete (solid lines; right image set) showing clearly that this ookinete exits the cell. The dashed and solid lines correspond to the section plane of the image sets. (b) An ookinete (green) emerging from the basolateral side of the invaded cell with its anterior end in the extracellular space (SS; empty arrowhead) and the posterior end inside the cell. The intracellular part of the ookinete is covered by a thin actin hood (arrow), best seen in the F-actin single channel image in the middle. The invaded cell is being protruded towards the midgut lumen (handlebar, *y*–*z* pseudo cross-section) as evidenced by the elevated basal cell surface. The position of the ookinete in respect to the epithelial cells is best viewed in the image on the right, which displays three sections arranged in their original relative orientations to give a pseudo-3D appearance simulating a slice as indicated by a blue line. Inset is a cartoon representation of the visible features. (c) A dead ookinete indicated by strong P28 staining but only remainders of GFP fluorescence, with its anterior end in association with the basolateral side of the invaded cell and fully surrounded by a thick ring-shaped F-actin hood (arrowhead). Note the signs of lysis at the anterior end (hollow arrowhead). The parasite is still inside the invaded cell and surrounded by a thinner actin hood than at the anterior end (arrows). Left panel is an overlay of all channels, while the right panel display F-actin only. (d) A dead ookinete (red) exiting the epithelium at the basolateral side of the invaded cell (*y*–*z* pseudo-cross-section) with its intracellular part covered by a distinct actin hood. Note the signs of lysis (solid arrowheads) and the thick ring-shaped F-actin hood (hollow arrowhead) at the posterior end in contrast to the parasite in c. The image on the left presents a more apical section (dashed lines), whereas the image on the right shows a more basal section (solid lines). Insets display individual P28 and F-actin channels.

In all recorded images, tissue polarity was clearly identifiable. The microvilli (MV)-rich lumen side of the epithelium usually exhibited a distinct and defined F-actin staining (Fig. 1, MV). This staining was frequently lost in cells that displayed a variety of the characteristics of invaded cells and was consistent with the documented loss of MV (data not shown). Actin layers that underlined plasma membranes sharply visualized the cell–cell borders that separate the invaded cell (Fig. 1, IC) from its neighbours (Fig. 1, NC), which became more diffuse towards the basal side reflecting the cavity-rich nature of the basal labyrinth. In addition, prominent staining of muscle fibres (Fig. 1, MF) that surround the midgut on its basal side was always detected, and further indicated the orientation of the epithelium. Exit of the ookinete from the basal side of the epithelium was mostly observed close but not limited to cell/cell borders.

Careful examination of serial confocal sections showed numerous ookinetes that appeared to exit the epithelium to be covered by a thin but well-defined layer of actin filaments (Fig. 1a, arrows). Among these, many events have been recorded suggesting that the actin-made hood is formed by the invaded cell itself. One example is presented in Fig. 1b, where only the apical tip of the invading parasite has exited the cell (Fig. 1b, arrowhead) and might have come in contact with neighbouring cells, while the peripheral part and the posterior end appear intracellular. The invaded cell is being protruded towards the lumen (Fig. 1b, handlebar in *y*–*z* pseudo-cross-section, cartoon in the right panel) and has formed a thin layer of actin filaments around the intracellular parts of the parasite (Fig. 1b, arrows).

Actin structures resembling hoods but not associated with live (GFP-positive) ookinetes were also frequently observed, although the surrounding areas displayed characteristics of apoptosis and midgut restitution. In these cases, actin hoods were thought to cover dead parasites that already have lost GFP fluorescence. To examine this, antibodies against the major ookinete surface protein P28 were used to distinguish living from dead ookinetes as introduced above, i.e. live parasites display dual fluorescence of GFP and anti-P28, while dead ookinetes display only P28 staining. Indeed, a large proportion of dead ookinetes, similar to the GFP-expressing parasites, exhibited an actin hood as they emerged from the epithelium (Fig. 1c). Morphological differences between parasite hoods around living or dead parasites were not detected; however, after carefully examining and comparing individual hooded ookinetes, it was noted that some parasites were complete engulfed in F-actin (Fig. 1d), while others displayed phalloidin staining mostly at one pole (Fig. 1c). Further, the intensity of staining, reflecting the density and thickness of the parasite hood, also varied among ookinetes (Fig. 1c, arrow versus arrowhead).

Despite morphological variability, the hood was always distinguishable from the actin underlining the plasma membrane of the epithelial cells in terms of location of the parasite and intensity of actin staining. Noteworthy, some invading parasites were heavily constricted (online Supplementary Material 1, arrow). This has been reported before for ookinetes crossing the epithelial cell membranes, and it was suggested that this is a type of parasite movement that facilitates transition through membranous barriers.^4^ However, we found that the majority of intracellular parasites that were constricted were also associated with actin filaments (online Supplementary Material 1, arrow). This was observed throughout the epithelial cells, even at the apical most side underneath the MV, and demonstrated a direct mechanical influence of the host cell actin cytoskeleton on malaria parasites. Like parasite hood, these actin filaments frequently were not associated with the typical actin distribution found in non-invaded cells, further indicating the delicate relation between invading ookinetes and the epithelial actin cytoskeleton.

### The parasite hood is actively formed around invading ookinetes

To examine whether the parasite hood is actively built around the parasite, antibodies against the *Drosophila melanogaster* WASP,^16^ a promoter of actin polymerization, were used in combination with phalloidin staining to identify the spatial distribution of the mosquito protein in relation to the actin cytoskeleton by confocal microscopy.

To estimate antibody specificity and ensure against non-specific reactivity of the anti-*Dm*WASP antibody in mosquitoes, we initially performed multiple alignment of the WASP peptide sequences of *A. gambiae* (AGAP001081), *Drosophila melanogaster* (CG1520-PA), *Aedes aegypti* (AAEL013892-PA), and *Culex pipiens* (CPIJ006699-PA), and two human variants (AAH02961, NP_003932), which showed that the proteins are evolutionary well conserved (online Supplementary Material 2, arrow). Sequence similarity is especially high in regions of suggestive functional domains, which were identified by Pfam analysis (online Supplementary Material 2, arrow). Indeed, in consistence with the predicted MW of WASP, Western blot analysis identified a specific 34 kDa band in mosquito midgut extracts which was absent in the RNAi knocked-down (kd) midguts, which were used as a control (online Supplementary Material 2, arrow).

**Figure 2:**
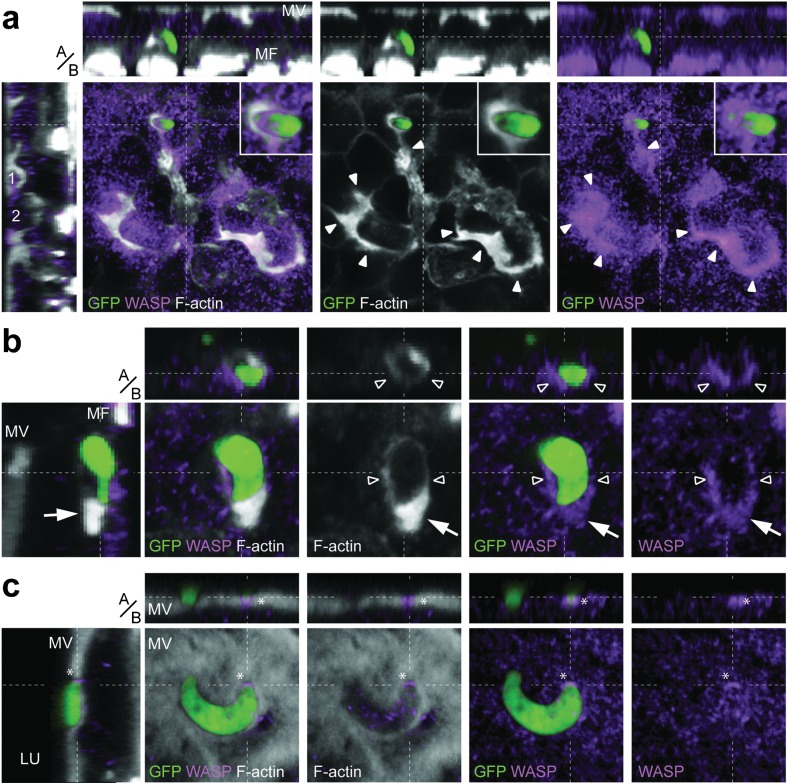
The F-actin rich hood is formed by the invaded cell. Panels show representative images including single confocal sections in the *x*–*y* plane, and pseudo-cross-sections in the perpendicular (apical, basal, a/b) *z*-axis, which are *x*–*z* (top) and *y*–*z* (left). *y*–*z* pseudo-cross-section is presented as channel overlay, while *x*–*y* section and *x*–*z* pseudo-cross-sections are presented as overlay and individual channels. The *x*- and *y*-axes are always in the plane of the epithelium. The position of the pseudo-cross-sections is indicated in the main images by dashed white lines; similarly, the plane of the main image is indicated by a dashed white line in each pseudo-cross-section. Parasite (green); F-actin staining with phalloidin (grey); WASP immunostaining (purple); microvilli (MV); muscle fibres (MF); cells^1^,^2^ protruded towards the midgut lumen (LU). Scale bars represent 5 *μ*m. (a) An ookinete exiting the epithelium. While the distribution of WASP staining is broader and more diffuse than the F-actin staining, WASP is co-localized with both, the parasite hood (inset), indicating that the hood is actively formed around the parasite, and the invaded areas showing extensive actin reorganization, which occurs during epithelial restitution (solid arrowheads). Note that *y*–*z* pseudo-cross-sections further show the ongoing restitution mechanisms in form of two cells that are getting extruded towards the midgut lumen (1 and 2), while the *x*–*z* pseudo-cross-section highlights the location of the hooded ookinete in the epithelium. While only one parasite is presented, two ookinetes were detected in the invaded area that both may contribute to the observable midgut restitution. (b) A hooded ookinete exiting the epithelium as indicated in the *y*–*z* pseudo-cross-section. The strong and massive actin hoods at the anterior end (arrow), as well as the thinner actin layers around the half-anterior part of the ookinete (hollow arrowheads), are co-localized with WASP. (c) Apically located non-hooded ookinete entering the epithelium showing WASP staining at its anterior end (asterisk).

WASP staining exhibited a wide and diffuse distribution in the epithelial cells (Fig. 2a). This staining was often weaker at the luminal side, and stronger towards the basal side, but it was most intense in invaded cells. In wounded and reconstituted areas that displayed extensive actin reorganization, WASP was co-localized with F-actin (Fig. 2a, arrow heads). Importantly, WASP was also co-localized with actin hoods of exiting parasites, indicating that the hood is actively formed around ookinetes during emergence from the epithelium (Fig. 2a, inset; Fig. 2a, arrow and hollow arrow heads). However, not all parasites were associated with WASP staining: while hooded parasites were rarely observed without WASP co-localization, non-hooded parasites were both WASP-positive and -negative (data not shown), further supporting a WASP-dependent, active formation of the hood. Even at the apical most side of the epithelium, WASP staining was observed to reflect the ookinete shape (Fig. 2c, asterisk).

### Silencing WASP increases hood formation

If the actin hood indeed is an epithelial defence reaction against invading ookinetes that ultimately determines parasite survival in the epithelium, induction of actin polymerization in the epithelium could be expected to lead to increased hood assembly that in turn inhibits parasite development. This hypothesis allows predicting that silencing an activator of actin polymerization, e.g. WASP, would result in a decrease of observable actin hoods.

**Figure 3:**
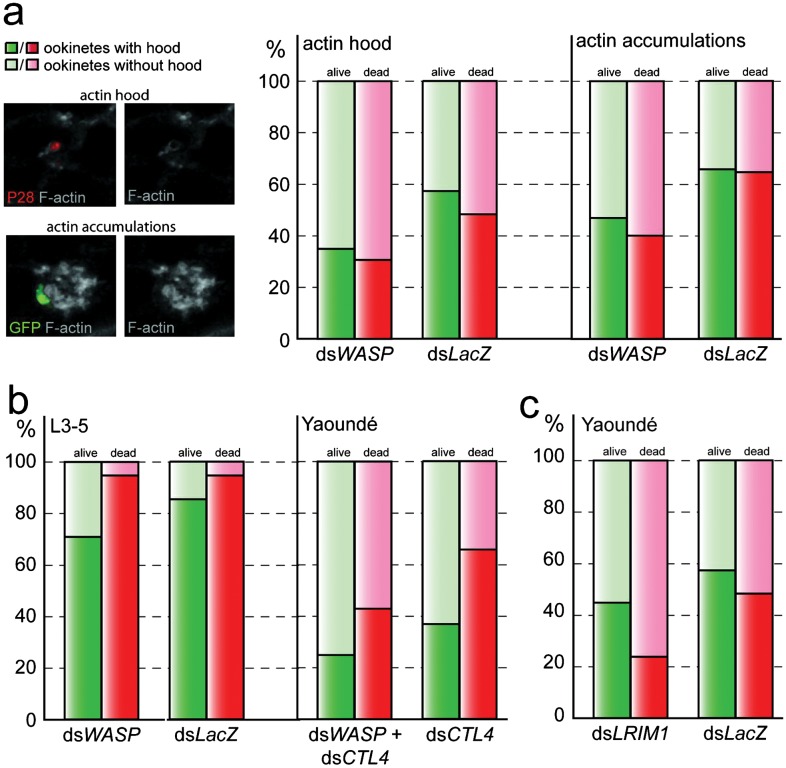
Impact of *WASP* gene silencing on epithelial actin cytoskeleton structure in respect to the invading ookinete in various mosquito genetic backgrounds. Graphs show occurrences (%) of discrete parasite hoods or diffuse actin accumulations in *Pb*GFP_CON_-infected midgut epithelia at 23–25 hours post-blood meal. Transgenic GFP expression in combination with anti P28-antibody staining was used to distinguish between live (GFP^+^P28^+^, green) and dead (GFP^−^P28^+^, red) parasites, while F-actin was visualized by phalloidin staining (grey). Representative images (a, left) of channel overlays or single channels (as indicated), from 3D projections of confocal sections showing the parasite hood (top) around a dead ookinete (red) in comparison to the less defined accumulations of actin around a living parasite (green; bottom). Hood occurrences at live (green) and dead (red) parasites of *WASP* kd *A. gambiae* Yaoundé mosquitoes (a, right), *WASP* kd melanizing L3-5 mosquitoes (b, left) and *CTL4* kd (b, right) or *LRIM1* kd (c) Yaoundé mosquitoes.

To test this hypothesis, an extensive blind analysis of actin hood formation was performed, where occurrences of hoods around viable and dead parasites were quantified in dsLacZ-injected control mosquitoes and compared to mosquitoes depleted for WASP (Fig. 3a, right panel).

As an additional control and to obtain a more general estimate of the effect of *WASP* silencing on F-actin distribution in the invaded midgut epithelium, diffuse accumulations of actin that were in direct contact with the parasite were quantified in parallel (Fig. 3a, right panel). These actin accumulations were spacious and of less defined morphology than the actin hood, and thus were often interpreted as artefacts of epithelial restitution that nonetheless could directly interact with the parasite during its migration through the epithelium.

In ds*LacZ*-injected control mosquitoes, 59% of living ookinetes exhibited the parasite hood as compared to 35% in mosquitoes depleted for WASP mosquitoes. Similarly, the number of parasite hoods around dead ookinetes was also considerably decreased to 31% in *WASP* kd mosquitoes as compared to 49% in control mosquitoes (Fig. 3a, left panel; online Supplementary Material 3). Chi-square goodness-<2?show=[to]?>of-fit test identified the decrease between both groups as significant (alive: *P * =  0.005; dead: *P*  =  0.008). These data further support the hypothesis that the parasite hood functions as an epithelial defence reaction.

Quantification of the less specific actin accumulations verified the regulatory role of WASP on the structure of the midgut epithelial actin cytoskeleton: actin accumulations were significantly decreased in the proximity of ookinetes in *WASP* kd mosquitoes (alive: 47%, *P*  =  0.001, dead: 40%, *P*  =  0.001) compared to the control group (alive: 65%, dead: 64%), regardless of the parasites’ state of health (GFP-positive or -negative).

### Killed ookinetes in L3-5 mosquitoes exhibit actin hoods

To investigate further the impact of the hood on parasite killing, hood occurrences around invading ookinetes were quantified in refractory *A. gambiae* L3-5 mosquitoes following silencing of *WASP* or injection with *dsLacZ* control (Fig. 3b, left panel). The L3-5 strain was genetically selected to be refractory to several *Plasmodium* species including *P. berghei*, by lysing a large fraction of the ookinetes and melanizing the remaining ones at the basal side of the midgut epithelium.^17^

In *dsLacZ*-injected control midguts, the majority (94–95%) of dead parasites were hooded with actin, suggesting that in L3-5 mosquitoes, the hood is directly or indirectly linked with ookinete killing. Furthermore, GFP-positive ookinetes which are likely at the start of their killing path also exhibited hoods more frequently in the L3-5 strain (86%) than in susceptible Yaoundé mosquitoes, suggesting that the hood may be directly involved in early stages of the killing reaction. Importantly, the hood frequency was significantly decreased to 71% (*P*  =  0.05) in *WASP* kd mosquitoes compared to controls (Fig. 3b, left panel; online Supplementary Material 3).

### Silencing WASP increases hood formation in CTL4 kd mosquitoes

To corroborate the above results and investigate further the putative involvement of WASP and the hood on parasite killing and melanization, an alternative refractoriness model was used,^10^ whereby silencing *CTL4* in susceptible mosquitoes leads to killing of the vast majority of ookinetes some directly through melanization.^18^ CTL4 is a C-type lectin with pleiotropic immune functions in *A. gambiae*, including negative regulation of the melanization reaction against *P. berghei* ookinetes.^10^,^19^

Mosquitoes of the susceptible Yaoundé strain were randomly divided into four equally sized groups. One group was concomitantly silenced for both *WASP* and *CTL4*, while other groups were injected either with *WASP*, *CTL4*, or control *LacZ* dsRNAs. Four independent biological replicates were performed (Fig. 3b, right panel; online Supplementary Material 3). Similar to hood occurrences in L3-5 mosquitoes, *WASP* kd decreased hood detection around live parasites in *CTL4* kd mosquitoes from 37% to 26%; however, this decrease was not statistically significant (*P*  =  0.113). On dead, GFP-negative, and P28-positive ookinetes, the hood decrease was more pronounced and statistically significant: from 67% in control *CTL4* kd to 44% in *CTL4*/*WASP* double kd mosquitoes (*P*  =  0.006), respectively.

### Silencing WASP rescues parasites in L3-5 and CTL4 kd mosquitoes

We examined in both L3-5 and *CTL4* kd mosquitoes whether silencing *WASP* not only reduced the hood occurrences, but also rescues any parasite numbers which would then develop to oocysts. The parasite density data were analysed by Mann–Whitney *U* test, while the Chi-square goodness-of-fit test was used to analyse putative differences in the ratio of melanized to live parasites between control and experimental mosquito groups.

**Figure 4:**
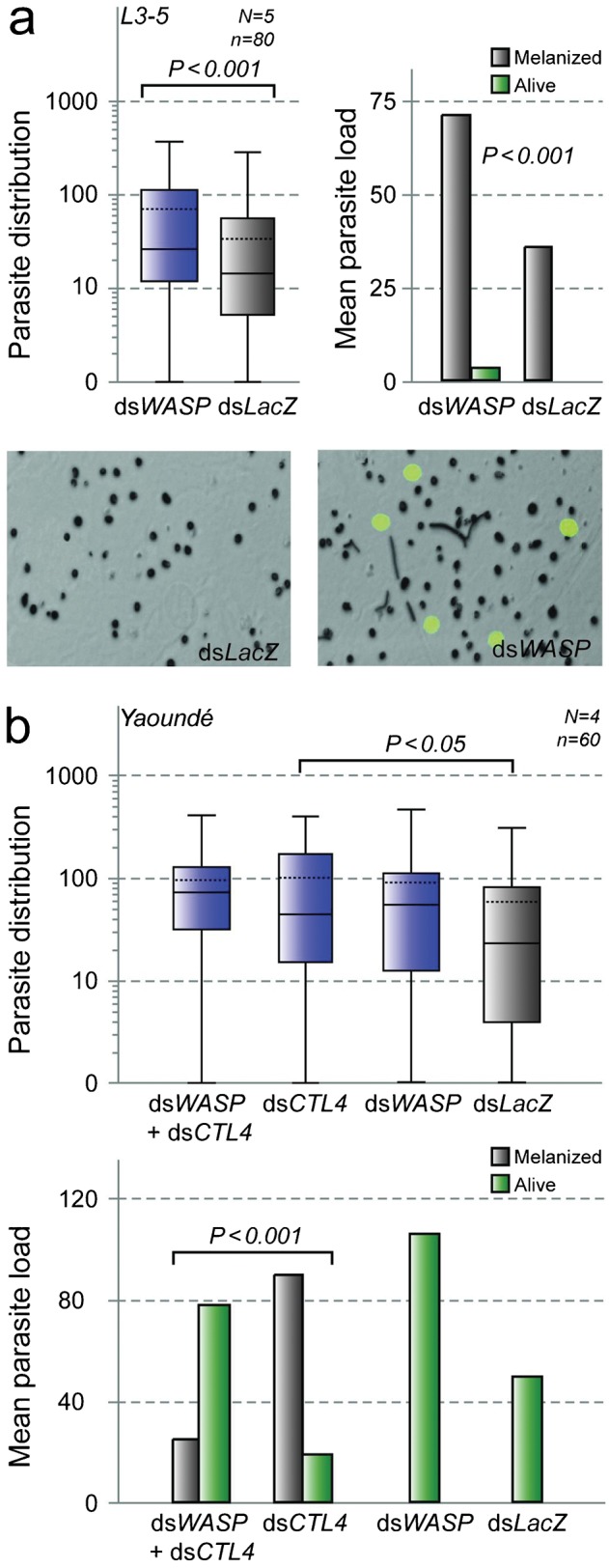
Effect of RNAi-mediated *WASP* gene silencing on *Plasmodium* density in refractory *A. gambiae* mosquitoes. Effects of RNAi-mediated *WASP* gene silencing on parasite development in two mosquito genetic backgrounds (a: L3-5; b: dsCTL4 Yaoundé). Distributions of total parasite numbers are presented as boxplots with mean (dotted lines) and median (solid lines) parasite densities indicated. The total number of midguts (*N*), the number of replicate experiments (*n*), and significant *P*-values are shown. *P*-values were estimated using Mann–Whitney *U* test. Mean parasite load graphs show corresponding mean numbers and standard errors of observed melanized ookinetes (grey) and live oocysts (green) as well as *P*-values determined by Chi-square goodness-of-fit test. (a) *WASP* gene silencing in the genetically selected L3-5 strain (Collins *et al.*, 1986). Note that *WASP* kd leads to increased numbers of melanized parasites, as well as significantly increased oocyst development. Representative images showing the effect of *WASP* silencing (ds*WASP*) on melanized *P. berghei* ookinetes, as well as live oocysts as compared to corresponding control (ds*LacZ*) mosquitoes. (b) Effects of *WASP* gene silencing on *P. berghei* in Yaoundé mosquitoes on a melanizing *CTL4* kd background. Importantly, the ratio of melanized parasites versus developing oocysts in *CTL4* kd mosquitoes is reversed after additional silencing of *WASP*.

In L3-5 mosquitoes, silencing *WASP* resulted in a significant increase of total parasite counts (melanized ookinetes plus oocysts) by 206% (*P*  =  0.04) compared to control (Fig. 4a, left). Among these, the number of melanized parasites was increased by 197% (Fig. 4a, right). Strikingly, although oocysts formed very rarely in control mosquitoes, *WASP* depletion allowed oocyst development in about 50% of *WASP* kd mosquitoes (online Supplementary Material 4) transforming them into potentially permissive vectors.

In the *CTL4* kd refractory system, in accordance with the L3-5 data, concomitant silencing of *WASP* and *CTL4* significantly reversed the *CTL4* kd phenotype (*P* > 0.001, Chi-square goodness-of-fit) allowing > 70% of the parasites to successfully develop to oocysts, in contrast to mosquitoes of the *CTL4* kd group that melanized about 75% of the observable parasites (Fig. 4b and online Supplementary Material 4). Melanization in *WASP* kd and ds*LacZ*-injected mosquitoes was negligible. The results from both the genetically selected (L3-5) and the epigenetically modified (*CTL4* kd) refractory systems suggest an essential role of *WASP* in parasite killing and corroborate the hypothesis of involvement of actin polymerization, probably leading to the actin hood, in the killing processes.

### The hood is linked to parasite lysis

**Figure 5:**
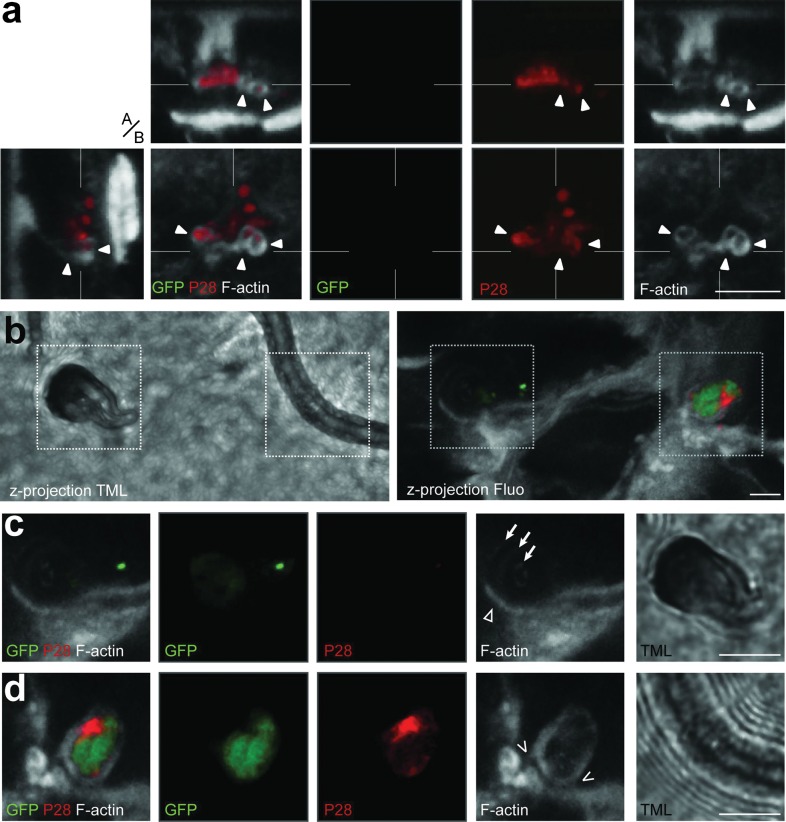
Localization of the actin hood during killing, lysis and melanization. Parasites at the midgut epithelium in refractory *A. gambiae* mosquitoes (A: L3-5; B: ds*CTL4* Yaoundé). Individual channels or channel overlay as indicated. Scale bars represent 5 *μ*m. (a) A dead ookinete at the basolateral side of the epithelium in L3-5 mosquitoes. The ookinete has lost its characteristic banana shape and displays a bleb-like morphology. Importantly, the lysis blebs are individually associated with thick ring-shaped F-actin hoods. Note the similarities between instances presented in Fig. 1c and d, where ring-shaped actin hoods are associated with parasite apical tip or posterior tip exhibiting signs of lysis. Presented are single confocal sections in the *x*–*y* plane with corresponding pseudo-cross-sections in the perpendicular (apical, basal, a/b) *z* axis (A), which are *x*–*z* (top) and *y*–*z* (left). The position of the pseudo-cross-sections is indicated in the main images by fine white lines; similarly, the plane of the main image is indicated by white lines in the pseudo-cross-sections. (b) Two ookinetes at the midgut epithelium in melanizing *CTL4* kd mosquitoes. Presented are *z*-projections of transmission light (TML; left) and fluorescence channel overlays (Fluo; right), as well as of the parasites. (c) Magnified individual confocal planes of the first ookinete which is getting melanized and this process seems not be completed yet, as melanin at the apical tip of the parasite appears thin and translucent as opposed to a thick melanin layer on the posterior end. Also weak GFP fluorescence can be detected, indicating that the ookinete only recently died. Note that P28 is not detected as melanin is impenetrable for antibodies. (d) Magnified individual confocal planes of the second ookinete shows no traces of melanization but is covered by an actin hood.

During careful analysis of our confocal data, we observed that P28-positive but GFP-negative parasites were frequently observed not exhibiting the banana shape that is characteristic for ookinetes but a bleb-like morphology that appeared suggestive of significant parasite destruction, presumably caused by lysis. The more advanced the destruction, the less recognizable the typical ookinete shape. In what we interpreted as one of the last observable phases of this lytic process, only fragments were detected that were P28-positive, while the surrounding epithelium showed characteristic signs of invasion. Importantly, many of these parasites exhibited strong association with F-actin; often, single lysis blebs were individually covered by actin (Fig. 5a). However, while the frequency of such observations differed and appeared to be more abundant in the L3-5 strain, no morphological differences of the parasites and the associated actin hoods were observed between *wt*, ds*CTL4*-injected Yaoundé mosquitoes and L3-5 mosquitoes. Our findings strongly suggested involvement of the hood in parasite killing/lysis.

Actin hoods were rarely observed to be associated with melanized parasites. In both L3-5 and *CTL4* kd Yaoundé mosquitoes, fully melanized ookinetes appeared mostly in the subepithelial space without visible physical connection to the epithelium. Owing to the impenetrable and light absorbing character of the melanin capsule, these ookinetes typically lacked anti-P28 and phalloidin staining and were mainly visible in transmission light microscopy. In contrast, ookinetes in the process of being melanized were often in contact with the epithelial cells and could often be visualised by their remaining GFP fluorescence and anti-P28 staining. However, usually actin hoods were observed at neither type of parasite. It was reported previously that melanized ookinetes exhibit organelle free actin zones (AZ), formed by cells neighbouring the invaded ones, sometimes even seen as alternating layers of actin and melanin.^14^ In our study, parasite-surrounding actin was only observed very rarely and at ookinetes that were not fully melanized (Fig. 5c, arrows). In contrast, AZ staining was often faint and appeared to be associated to the typical actin cytoskeleton morphology, e.g. in the form of connections to putative cell/cell borders (Fig. 5c, open arrow head), which is in accordance with the proposed origin of this structure from neighbouring cells. Direct comparisons of the AZ with the hood, whenever the two types were co-recorded in the same 3D confocal image stack, revealed that the hood is a much thicker structure, stained stronger with phalloidin, and that in contrast to the AZ, it frequently appeared not to be associated with any other actin-stained cellular structure (Fig. 5d).

### LRIM1 is involved in hood-linked parasite killing

**Figure 6:**
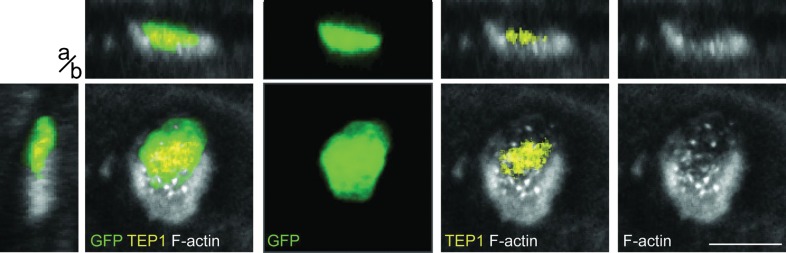
The actin hood and TEP do not co-localize. A live ookinete is exiting the invaded midgut epithelial cell and is covered by an actin hood (grey). Co-localization of TEP1 (yellow) and F-actin is not apparent: the hood is the strongest on the lateral parasite side towards to the basal side of the epithelium; in contrast, TEP1 is localized at the middle of the parasite. Presented are single *x*–*y* sections in the plane of the epithelium (large panels), as well as pseudo-cross-sections in the perpendicular (apical, basal, a/b) z axis, which are *x*–*z* (top) and *y*–*z* (left). Individual channels and channel overlays as indicated. Scale bar represents 5 *μ*m.

The complement cascade is largely responsible for the observed ookinete killing in the midgut epithelium.^7^,^10^,^20^,^21^ Silencing of the complement factors TEP1, LRIM1, or APL1C rescues the majority of parasites that are otherwise cleared and appear as GFP-negative using the assay described above. The same cascade is central in bacterial phagocytosis.^8^,^9^ To further elucidate the role of the actin hood in the defence against invading parasites, the distribution of the complement effector TEP1 on invading ookinetes at 23–25 hours after the infectious blood meal was compared with that of F-actin. While TEP1 and the hood were frequently associated with the same parasites, direct co-localization of the two proteins was never observed (Fig. 6). Indeed, TEP1 was usually localized on the parasite, while the hood surrounded the parasite and TEP1 staining. These data suggested that the formation of the hood and the complement initiation on the parasites may cooperate towards parasite killing.

We investigated the connection between these two mechanisms by silencing *LRIM1* and quantifying the number of hoods (Fig. 3c and online Supplementary Material 3). Ds*LacZ*-injected mosquitoes were used as controls. While only minor differences (*P*  =  0.197) in the frequency of parasite hoods around living ookinetes were observed between *LRIM1* kd (46%) and control mosquitoes (59%), significantly less hoods were observed around dead ookinetes in *LRIM1* kd mosquitoes (24%) compared to the control group (49%, *P*  =  0.004). These data suggest a functional link between these two processes, which culminate with ookinete killing.

## Discussion

The cellular and molecular interactions between mosquitoes and parasites during the ookinete-to-oocyst transition have received considerable attention in recent years. The reorganization of the actin cytoskeleton has been studied in the context of the damage inflicted to the invaded cell and the subsequent epithelial restitution, and only few studies have examined the impact of the actin cytoskeleton on the invading parasite. A previous *in vivo* imaging analysis of midgut invasion by *P. berghei* revealed an actin-based membranous structure, termed ‘hood’ for its morphological appearance, to surround ookinetes as they exit the epithelial cell layer.^4^ Further, a fibrillar organelle-free zone was reported to surround *P. gallinaceum* ookinetes in a refractory, lytic strain of *A. gambiae*.^13^ This, together with the morphological resemblance of the parasite hood with the phagocytic cup and the involvement of the mosquito immune reactions in phagocytosis, as well as in defence against parasites, i.e. those involving the complement factors LRIM1 and TEP1,^4,7^–^10^ suggested that the parasite hood might represent a local epithelial defence reaction. Support for this theory came from our previous study, were we showed that depletion of positive regulators of actin polymerization increased *P. berghei* oocyst density, while depletion of negative regulators decreased parasite populations.^11^,^12^

Here, using the F-actin marker phalloidin and confocal microscopy, we show that the parasite hood is an actin-rich structure formed by the invading cell as the ookinete leaves the cell exiting into the sub-epithelial space. Using antibodies against WASP, an activator of actin filament nucleation, we show that the hood is the result of signaling initiated on the parasite surface. This may be triggered upon contact of the parasite with the basolateral side of the invaded cells, as an epithelial defence mechanism to decelerate parasites, prolonging their exposure to the fatal environment of the apoptotic cell. However, the location of the hood at the basal side of the epithelium where the parasite comes into contact with the hemolymph, the bearer of the humoral immune reactions, may suggest an additional or alternative hypothesis. Indeed, the link between the complement mediated killing of parasites and the formation of the hood revealed here indicate that the hood may provide a structural interface for the complement cascade to attack the invading parasites, while at the same time, it appears to follow the initiation of the complement reaction, perhaps as a disposal mechanism of dying parasites.

In this context, it is worth highlighting the parallel between the parasite hood and phagocytosis, where WASP is shown to be a key regulator of the formation of the phagocytic cup.^22^ While no immediate co-localization between the hood and TEP1 was observed, TEP1 staining was frequently found on dead hooded parasites. Furthermore, silencing of *LRIM1* that leads to perishing of the cleaved TEP1_cut_, a TEP1 form that can initiate complement reactions on the microbial surfaces,^23^ leads to less dead hooded ookinetes. At the same time the number of hooded living ookinetes does not differ between *LRIM1* kd and control mosquitoes, suggesting that complement activation may be connected with the parasite hood. It is tempting to speculate that the hood is formed following complement activation on the parasite surface and similar to the formation of the phagosome, is serving as the ‘reactive site’ for parasite killing through the attack by reactive oxygen species and other effectors. As invaded cells undergo programmed cell death and are expelled towards the midgut epithelium, dead ookinetes are presumably fully engulfed by the hood and are eliminated during that process.

An alternative hypothesis for the function of the hood is that it facilitates the ookinete exit from the epithelial cell. In this scenario, the parasite may require the hood as part of an exit mechanism, or the midgut cell may require the hood to prevent cytoplasm draining into the haemocoel through the exit site. For the former, it is plausible that the parasite induces reorganization of the host cell actin cytoskeleton that results in the formation of the hood. Subversion of the host cell cytoskeleton is a virulence strategy that is employed by many bacterial pathogens. The *Citrobacter rodentium* protein EspT activates host cell Rac-1 and Cdc42 and thereby, triggers the formation of lamellipodia and membrane ruffles through WASP and the Arp2/3 pathway.^24^ Also *Salmonella* orchestrates the modulation of Rho family GTPase activity in the host cell that triggers localized remodelling of the actin cytoskeleton.^25^ Most importantly, *P. berghei* sporozoites appear to utilize the host cell actin cytoskeleton to form a thin sheath of F-actin that covers them during entrance in red blood cell-like cells.^26^ In another *Apicomplexa*, *Toxoplasma gondii*, entrance into HeLa cells is accompanied by local actin reorganization in the host cell that forms a ring structure around the pathogen,^26^ and the Arp2/3 complex has been shown to localize with the F-actin ring. However, this hypothesis of parasite-induced actin remodelling fails to explain the observed RNAi phenotypes. WASP depletion increases the number of melanized parasites in the refractory L3-5 strain, while in parallel allowing sparse oocyst development. In contrast, concomitant kd of WASP and CTL4 in susceptible mosquitoes inverts the melanized/alive parasite ratio of *CTL4*-only kd mosquitoes, by allowing development of 80% of the observed parasites. It has been previously proposed that L3-5 refractoriness kills parasites with subsequent clearance by lysis or melanization, whereas *CLT4*-depleted Yaoundé mosquitoes melanize and thereby kill ookinetes.^7^,^18^ In this context, the actin cytoskeleton of the mosquito midgut epithelium has been morphologically linked to parasite melanization.^14^ Besides a local epithelial response to the invading parasite, here systemic responses may also explain many of the observations. Silencing of WASP may impair vesicular-based secretion of hemolymph circulating immune factors by the fat body and, as a consequence, systemic immune responses can be compromised. While this hypothesis plausibly accounts for the increased survival of parasites after depletion of *WASP* in wild-type and *CTL4* kd Yaoundé mosquitoes, it cannot explain the increased number of melanized parasites in the L3-5 strain following *WASP* kd. Therefore, the most parsimonious explanation at the moment is that the hood is required for parasite killing and lysis and that its depletion through *WASP* kd allows more parasites to escape this reaction, which subsequently become melanized in the sub-epithelial space L3-5 mosquitoes. A small fraction of these parasites escape melanization and develop to oocysts.

An alternative or complementary explanation may be that *WASP* kd affects migration of and immune protein secretion by hemocytes, including the complement and melanization cascade mediators TEP1, LRIM1 and phenoloxidase, thus compromising the ability of mosquitoes to defend against parasites. Indeed, a recent study revealed that hemocytes undergo significant migration following infection.^27^ However, the melanization reaction seems to remain intact in *WASP* kd mosquitoes; therefore, this hypothesis is less likely.

While the experiments reported here were performed using the convenient *P. berghei*/*A. gambiae* system, we have previously shown that the importance of WASP, and presumably the hood, is not limited to the mouse malaria transmission model. It also applies to sympatric infections of mosquitoes with field isolates of the major human malaria parasite *P. falciparum*. Therefore, understanding the mechanism of these important vector responses is expected to educate our efforts to develop new and robust methods to eliminate the disease.

## Material and Methods

### *A. gambiae* rearing and infections with *P. berghei*

Mosquitoes of the *A. gambiae* strain Yaoundé^28^ and L3-5^17^ were reared at 27°C with a relative humidity of 75% and a 12/12 hour light/darkness cycle. All infection experiments were performed using the *Pb*GFP_CON_ transgenic 259cl2 strain.^15^ Mosquito infections were performed as described.^12^ Fed mosquitoes were maintained at 19°C, while unfed mosquitoes were removed from the colony 28 hours post-infectious blood meal. All procedures involving animal work were carried out with licenses from the respective institutions and under the strictest ethical criteria.

### DsRNA-mediated gene silencing in adult female *A. gambiae*

Production of dsRNA and mosquito gene silencing were carried out as described previously.^7^,^10^ Primer sequences for dsRNA production were, without T7 sequences, 5′-GGTGGTGCAGCTGTACACGA-3′ and 5′-TGGGCCGTCTGTAGTGGAAA-3′ for *WASP*, 5′-AATATCTATCTCGCGAACAATAA-3′ and 5′-TGGCACGGTACACTCTTCC-3′ for *LRIM1*, 5′-TGGTTTGATGCCGTGTCCT-3′ and 5′-AATAAATTGTCTCGGTTCATCATC-3′ for *CTL4*, and 5′-AGAATCCGACGGGTTGTTACT-3′ and 5′-CACCACGCTCATCGATAATTT-3′ for exogenous *LacZ*. Gene kd efficiency was analysed by quantitative real-time PCR as described elsewhere^11^ using the primers 5′-TGTCTCAAGGCGAACCAGATG-3′ and 5′-TTGCGTCACCTCGATCTTCTC-3′ for WASP, 5′-TGTCTCAAGGCGAACCAGA TG-3′ and 5′-TTGCGTCACCTCGATCTTCTC-3′ for *LRIM1*, and 5′-GTGCGCGAGTTGGAGAAGA-3′ and 5′-ATCGGTTTGGGCAGAATGC-3′ for S7, which was used to normalize data. Only experiments where kd efficiency ranged above 50% were included in data analysis. Significance of total parasite density differences between kd and control mosquitoes was analysed by Mann–Whitney *U* test; differences between melanized ookinetes versus live oocysts were analysed using the Chi-square goodness-of-fit test.

### Tissue staining and confocal microscopy

Mosquito midgut epithelia were dissected in ice-cold phosphate-buffered saline (PBS), opened longitudinally to remove midgut contents and incubated in fixative (4% formaldehyde, 1× PBS, 2 mM MgSO_4_, 1 mM EGTA) for 35–45 minutes. Fixation and all subsequent steps were performed at room temperature unless indicated otherwise. After fixation, midgut epithelial sheets were blocked for 90 minutes in 1% bovine serum albumin and 0.1% Triton X-100 in PBS (PBT).^23^ Antibody solutions were prepared in PBT with 1∶750 mouse anti-P28, 1∶1000 rabbit anti-DmWASP (gift from E. D. Schejter), and 1∶350 rabbit anti-AgTep1 and tissues were incubated over night at 4°C. Tissues that served as negative controls were incubated in blocking buffer without antibodies under the same conditions. Then samples were incubated for 1–3 hours in 1∶1500 dilutions of Alexa Fluor goat anti-mouse or 1∶3000 dilutions or Alexa goat anti-rabbit (both Molecular Probes, Eugene, OR, USA) in PBT. For actin staining, tissues were incubated with conjugated phalloidin dyes (Invitrogen, Carlsbad, CA, USA) for 20 minutes. After mounting tissues on microscope slides using VECTASHIELD Mounting Medium (Vector Labs, Burlingame, CA, USA), visualization was achieved on Leica SP5 confocal microscopes. Commonly, images were background-corrected and noise-filtered with the Leica LAS AF software (Leica Microsystems, Wetzlar, Germany). Additional image adjustments (sectioning, cropping, brightness/contrast adjustment) were performed with the Volocity (PerkinElmer Inc., Beaconsfield, UK) and Adobe Photoshop CS2 (Adobe, San Jose, CA, USA) software.

### Quantitative hood analysis

To quantitatively analyse the impact of *A. gambiae* gene silencing of candidates on formation of the parasite hood, RNAi-mediated kd and tissue staining was performed as described above. 3D confocal stacks were recorded on a Leica SP5 microscope (Leica, Wetzlar, Germany), while GFP-fluorescence and P28 staining but not actin staining were used to select areas of interest without introducing any bias due to the morphology of the actin cytoskeleton. Data sets from all gene kd and control groups from the various experiments were then pooled and experimental status (ds*WASP*, ds*LRIM1*, ds*LacZ*) was masked unrecognizable by denomination with random numbers. Recorded parasites were then evaluated for zonular actin staining (hood) and larger diffuse accumulations of actin using the Volocity software (PerkinElmer Inc.). The difference in occurrences of parasite hoods or actin accumulations between kd and their control mosquitoes were analysed, after gene kd group memberships were reassigned, with the Chi-square goodness-of-fit test.

### Multiple sequence alignment and Pfam analysis

Multiple sequence alignment was performed with WASP protein sequences from *A. gambiae* (AGAP001081-PA; VectorBase) and *D. melanogaster* (CG1520-PA; VectorBase), as well as the *Culicidae A. aegypti* (AAEL013892-PA; Vectorbase) and *C. quinquefasciatus* (CPIJ006699-PA; VectorBase) and two human WASP variants (AAH02961 and NP_003932; National Institute of Health, Bethesda, MD, USA) using the ClustalX Software version 2.0.12 for Mac. The Gonnet series protein weight matrix was used and each alignment step was iterated. Sequences obtained from VectroBase and NIH were obtained via http://www.vectorbase.org and http://www.ncbi.nlm.nih.gov, respectively. Pfam analysis with the given sequences was performed online at http://pfam.sanger.ac.uk.

### SDS–PAGE and Western blot analysis

For Western blot analysis of kd and control mosquitoes, tissues were collected 3 days post-dsRNA injection. The heads of seven mosquitoes per kd and control group were cut off on ice and the remaining carcasses were subjected to Ultrafree-MS 0.22 μm filter tubes (Millipore Corporation, Billerica, MA, USA), spun down for 5 minutes at 4°C and all liquids were collected in 0.25 M Tris pH 6.8, 40% glycerol, 8% SDS, and 8% b-mercaptoethanol. Proteins were resolved by discontinuous SDS–PAGE, with 12% resolving gel, and either subsequently transferred to Hybond-P membranes (GE Healthcare, Buckinghamshire, UK) in a Trans-Blot SD Semi-Dry Transfer Cell (BioRad, Hercules, CA, USA) blotting chamber for subsequent Western Blot analysis or stained with Imperial^TM^ Protein Stain (Pierce, Rockford, IL, USA). Blocking of membranes was performed o/n at 4°C in 1% Tween, and 3% Milk powder in PBS (blocking buffer). Subsequently, membranes were incubated for 2 hours with 1∶1000 dilution of rabbit anti-WASP (gift from E. J. Schejter) and 1∶1000 of rabbit anti-Tubulin (Sigma-Aldrich, Suffolk, UK), followed by 1–3 hours of incubation with 1 ∶30 000 HRP-conjugated goat anti-rabbit antibodies (Promega, Southampton, UK). All antibody incubations were performed at room temperature in blocking buffer. Detection was performed using Western Lightning Chemiluminescence Reagent Plus Kit (PerkinElmer Life Sciences, Waltham, MA, USA).

## References

[1] Sinden RE (2002). Molecular interactions between *Plasmodium* and its insect vectors.. Cell Microbiol..

[2] Baton LA, Ranford-Cartwright LC (2004). *Plasmodium falciparum* ookinete invasion of the midgut epithelium of *Anopheles stephensi* is consistent with the Time Bomb model.. Parasitology.

[3] Gupta L, Kumar S, Han YS, Pimenta PF, Barillas-Mury C (2005). Midgut epithelial responses of different mosquito–*Plasmodium* combinations: the actin cone zipper repair mechanism in *Aedes aegypti*.. Proc Natl Acad Sci USA..

[4] Vlachou D, Zimmermann T, Cantera R, Janse CJ, Waters AP, Kafatos FC (2004). Real-time, *in vivo* analysis of malaria ookinete locomotion and mosquito midgut invasion.. Cell Microbiol..

[5] Meis JF, Pool G, van Gemert GJ, Lensen AH, Ponnudurai T, Meuwissen JH (1989). *Plasmodium falciparum* ookinetes migrate intercellularly through *Anopheles stephensi* midgut epithelium.. Parasitol Res..

[6] Han YS, Thompson J, Kafatos FC, Barillas-Mury C (2000). Molecular interactions between *Anopheles stephensi* midgut cells and *Plasmodium berghei*: the time bomb theory of ookinete invasion of mosquitoes.. EMBO J..

[7] Blandin S, Shiao SH, Moita LF, Janse CJ, Waters AP, Kafatos FC (2004). Complement-like protein TEP1 is a determinant of vectorial capacity in the malaria vector *Anopheles gambiae*.. Cell..

[8] Levashina EA, Moita LF, Blandin S, Vriend G, Lagueux M, Kafatos FC (2001). Conserved role of a complement-like protein in phagocytosis revealed by dsRNA knockout in cultured cells of the mosquito, *Anopheles gambiae*.. Cell..

[9] Moita C, Simoes S, Moita LF, Jacinto A, Fernandes P (2005). The cadherin superfamily in *Anopheles gambiae*: a comparative study with *Drosophila melanogaster*.. Comp Funct Genomics..

[10] Osta MA, Christophides GK, Kafatos FC (2004). Effects of mosquito genes on *Plasmodium* development.. Science..

[11] Mendes AM, Schlegelmilch T, Cohuet A, Awono-Ambene P, de Iorio M, Fontenille D (2008). Conserved mosquito/parasite interactions affect development of *Plasmodium falciparum* in Africa.. PLoS Pathog..

[12] Vlachou D, Schlegelmilch T, Christophides GK, Kafatos FC (2005). Functional genomic analysis of midgut epithelial responses in Anopheles during *Plasmodium* invasion.. Curr Biol..

[13] Vernick KD, Fujioka H, Seeley DC, Tandler B, Aikawa M, Miller LH (1995). *Plasmodium gallinaceum*: a refractory mechanism of ookinete killing in the mosquito, *Anopheles gambiae.*. Exp Parasitol..

[14] Shiao SH, Whitten MM, Zachary D, Hoffmann JA, Levashina EA (2006). Fz2 and cdc42 mediate melanization and actin polymerization but are dispensable for *Plasmodium* killing in the mosquito midgut.. PLoS Pathog..

[15] Franke-Fayard B, Trueman H, Ramesar J, Mendoza J, van der Keur M, van der Linden R (2004). A *Plasmodium berghei* reference line that constitutively expresses GFP at a high level throughout the complete life cycle.. Mol Biochem Parasitol..

[16] Ben-Yaacov S, Le Borgne R, Abramson I, Schweisguth F, Schejter ED (2001). Wasp, the drosophila Wiskott–Aldrich syndrome gene homologue, is required for cell fate decisions mediated by Notch signaling.. J Cell Biol..

[17] Collins WE, McClure HM, Swenson RB, Mehaffey PC, Skinner JC (1986). Infection of mosquitoes with *Plasmodium vivax* from chimpanzees using membrane feeding.. Am J Trop Med Hyg..

[18] Volz J, Muller HM, Zdanowicz A, Kafatos FC, Osta MA (2006). A genetic module regulates the melanization response of *Anopheles* to *Plasmodium*.. Cell Microbiol..

[19] Schnitger AK, Yassine H, Kafatos FC, Osta MA (2009). Two C-type lectins cooperate to defend *Anopheles gambiae* against Gram-negative bacteria.. J Biol Chem..

[20] Fraiture M, Baxter RH, Steinert S, Chelliah Y, Frolet C, Quispe-Tintaya W (2009). Two mosquito LRR proteins function as complement control factors in the TEP1-mediated killing of *Plasmodium*.. Cell Host Microbe..

[21] Povelones M, Waterhouse RM, Kafatos FC, Christophides GK (2009). Leucine-rich repeat protein complex activates mosquito complement in defense against *Plasmodium* parasites.. Science..

[22] Tsuboi S, Meerloo J (2007). Wiskott–Aldrich syndrome protein is a key regulator of the phagocytic cup formation in macrophages.. J Biol Chem..

[23] Povelones M, Bhagavatula L, Yassine H, Tan LA, Upton LM, Osta MA (2013). The CLIP-domain serine protease homolog SPCLIP1 regulates complement recruitment to microbial surfaces in the malaria mosquito *Anopheles gambiae*.. PLoS Pathog..

[24] Bulgin RR, Arbeloa A, Chung JC, Frankel G (2009). EspT triggers formation of lamellipodia and membrane ruffles through activation of Rac-1 and Cdc42.. Cell Microbiol..

[25] Patel JC, Galan JE (2008). Investigating the function of Rho family GTPases during *Salmonella*/host cell interactions.. Methods Enzymol..

[26] Gonzalez V, Combe A, David V, Malmquist NA, Delorme V, Leroy C (2009). Host cell entry by apicomplexa parasites requires actin polymerization in the host cell.. Cell Host Microbe..

[27] King JG, Hillyer JF (2013). Spatial and temporal *in vivo* analysis of circulating and sessile immune cells in mosquitoes: hemocyte mitosis following infection.. BMC Biol..

[28] Tchuinkam T, Mulder B, Dechering K, Stoffels H, Verhave JP, Cot M (1993). Experimental infections of *Anopheles gambiae* with *Plasmodium falciparum* of naturally infected gametocyte carriers in Cameroon: factors influencing the infectivity to mosquitoes.. Trop Med Parasitol..

